# Evaluation of the VITEK^®^ MS PRIME system for routine identification of bacteria, yeasts, and molds in a tertiary care hospital laboratory

**DOI:** 10.1007/s10096-025-05386-0

**Published:** 2026-03-30

**Authors:** Carlotta Magrì, Elena De Carolis, Vittorio Ivagnes, Brunella Posteraro, Maurizio Sanguinetti

**Affiliations:** 1 Dipartimento di Scienze Biotecnologiche di Base, Cliniche Intensivologiche e Perioperatorie, Università Cattolica del Sacro Cuore, Rome, Italy; 2 Dipartimento di Scienze di Laboratorio ed Ematologiche, Fondazione Policlinico Universitario A. Gemelli IRCCS, Rome, Italy; 3 Direzione Scientifica, Unità Operativa “Medicina di Precisione in Microbiologia Clinica”, Fondazione Policlinico Universitario A. Gemelli IRCCS, Rome, Italy

**Keywords:** MALDI-TOF MS, VITEK MS PRIME, MALDI Biotyper, Bacteria, Yeasts, Filamentous fungi

## Abstract

**Purpose:**

Matrix-assisted laser desorption ionization–time of flight mass spectrometry (MALDI-TOF MS) is integral to routine microbial identification. This study examines the performance of the VITEK® MS PRIME (bioMérieux; “PRIME”) system for identification of clinically relevant bacteria, yeasts, and molds. Methods: We evaluated PRIME using the in vitro diagnostic (IVD) knowledge base version 3.2 (KB3.2) database on 652 clinical isolates, with reference identifications by polymerase chain reaction (PCR) and DNA sequencing. We also compared PRIME results with those obtained using the MALDI Biotyper (Bruker Daltonics; “Biotyper”) for bacteria and yeasts in our routine workflow.

**Results:**

PRIME correctly identified 311/317 bacteria (98.1%), 185/196 yeasts (94.4%), and 112/130 molds (86.2%); non-identification for molds was 16/130 (12.3%), with 2/130 (1.5%) misidentifications. Biotyper achieved 308/317 (97.2%) correct identifications for bacteria and 120/196 (61.2%) for yeasts with the IVD database; using the research use only (RUO) database increased the identification rate to 184/196 (93.9%) and reduced non-identifications from 76/196 (38.8%) to 12/196 (6.1%) for yeasts. Misidentifications by PRIME were uncommon, whereas molds—assessed only with PRIME—showed more frequent non-identification and occasional cross-taxon assignments. For example, *Fusarium solani* was reported as *Rhizopus arrhizus*, reflecting persistent challenges in MALDI-TOF MS for filamentous fungi and the impact of database breadth. Notably, mold performance observed here lies near the upper range of recent PRIME evaluations using IVD KB3.2.

**Conclusions:**

Overall, PRIME provided reliable routine identification for bacteria, yeasts, and molds; further expansion of fungal reference spectra and optimization of extraction workflows should improve mold identification. Pending such improvements, possible non-identifications or discordant assignments should be adjudicated by conventional microbiology before reporting.

**Supplementary Information:**

The online version contains supplementary material available at 10.1007/s10096-025-05386-0.

## Introduction

Matrix-assisted laser desorption ionization–time of flight mass spectrometry (MALDI-TOF MS) has revolutionized clinical microbiology by enabling rapid and reliable identification of a wide range of microorganisms [15, 16]. Timely and accurate species identification is critical for clinical decision-making, as it informs the initiation and optimization of antimicrobial therapy and directly influences patient outcomes [19]. Since its introduction into routine diagnostics, two systems have become widely adopted: the MALDI Biotyper system (Bruker Daltonics, Bremen, Germany) and the VITEK^®^MS system (bioMérieux, Marcy l’Étoile, France). Both have shown excellent performance [10–12, 17], though they differ in database structure, user interface, spectrum analysis algorithms, and sample preparation methods [13].

The VITEK^®^ MS PRIME (“PRIME”) system is the most recent MALDI-TOF MS platform developed by bioMérieux. It features several workflow improvements, including automated continuous target slide processing (“load and go” with a capacity up to 16 slides) and urgent slide prioritization. Importantly, it also supports the identification of filamentous fungi through dedicated databases available in both in vitro diagnostic (IVD) and research use only (RUO) formats.

Recent studies have reported encouraging results for PRIME. Bosserman et al. [2] evaluated PRIME using the IVD KB3.2 database in a high-throughput North American clinical laboratory, focusing on bacteria and yeasts, and showed comparable identification rates and shorter hands-on time with PRIME than with Biotyper. Lee et al. [9] evaluated the system’s performance on clinical isolates of filamentous fungi, highlighting the added value of the RUO database, especially for accurate identification of basidiomycetes. More recently, Dutkiewicz et al. [6] and Klugherz et al. [7] evaluated PRIME in the context of fungal identification using the IVD KB3.2 database, reporting species-level identification rates in the range of approximately 77–81% across panels enriched for uncommon yeasts, *Aspergillus* spp., and rare filamentous fungi, or including a more evenly distributed mold set typical of clinical mycology. Klugherz et al. [7], in a head-to-head comparison of three MALDI-TOF MS systems, found that PRIME failed to identify a greater number of clinical filamentous fungal isolates.

Despite these findings, independent evaluations of PRIME in routine European tertiary-care settings remain limited. In particular, no study has yet assessed PRIME with the IVD KB3.2 database across bacteria, yeasts, and molds within a single, broad isolate panel representative of daily diagnostic activity.

In this study, we evaluated the VITEK^®^ MS PRIME system for the identification of bacteria, yeasts, and molds in a routine clinical microbiology laboratory in a European tertiary-care hospital. A broad and taxonomically diverse panel of clinical isolates, previously identified by PCR and sequencing, was analyzed using the IVD KB3.2 database. We specifically aimed to assess whether PRIME could serve as a single MALDI-TOF MS platform for routine identification of bacteria, yeasts, and molds in this setting and to contextualize its performance against the MALDI Biotyper (“Biotyper”) system for bacterial and yeast isolates.

## Methods

### Study design and samples

A total of 652 clinical isolates were analyzed, including 319 Gram-positive and Gram-negative bacteria, 200 yeasts, and 133 filamentous fungi, with one isolate per patient per species. Isolates were obtained either from routine microbiological diagnostics performed in 2024 at the clinical microbiology laboratory of the Fondazione Policlinico Universitario “Agostino Gemelli” IRCCS in Rome, Italy, or from the laboratory’s frozen clinical collection, stored at − 80 °C in glycerol. All isolates were tested retrospectively for the purpose of this study. The isolate set included a wide range of microbial genera and species, derived from various clinical sample types, primarily blood and respiratory specimens.

For study purposes, all isolates regardless of their original routine identification—were re-identified using established PCR and sequencing methods, and this identification was used as the reference standard for performance analyses (see below).

Stored isolates were revitalized by subculturing on appropriate agar media prior to testing. Growth and/or subculture were performed on the following media: blood agar (tryptic soy agar with 5% sheep blood or Schaedler blood agar), chocolate PolyViteX (PVX) agar, or MacConkey agar (all from bioMérieux) for Gram-positive and Gram-negative bacteria; Candida BromoCresol Green (BCG) agar (KIMA, Piove Di Sacco, Padua, Italy) for yeasts; and Sabouraud dextrose agar (Liofilchem, Roseto degli Abruzzi, Teramo, Italy) for filamentous fungi. Bacterial and yeast isolates were incubated at 37 °C for 24 to 48 h, while filamentous fungi were incubated at 30 °C for a minimum of 48 h and up to 5 days, depending on growth characteristics. Incubation under 5% CO_2_-enriched or anaerobic conditions was applied as needed to support the growth of fastidious aerobic or anaerobic bacteria, respectively.

## PRIME system-based analysis

Mass spectra were acquired using the VITEK^®^ MS PRIME system (bioMérieux) in automatic mode, with a laser frequency of 1000 Hz and in positive linear mode, following the manufacturer’s instructions for IVD use. For bacterial and yeast samples, a small amount of colony was collected directly from the agar plate using a PICKME pen and transferred to a VITEK MS-DS target plate, with each isolate spotted once. For yeast samples, 1 µL of 70% formic acid was added to the spot. After air drying, 1 µL of α-cyano-4-hydroxycinnamic acid (CHCA) matrix solution (prepared in 50% acetonitrile and 2.5% trifluoroacetic acid) was applied to both the sample and calibration spots. The target plate was allowed to air dry before being loaded into the instrument.

Filamentous fungal samples were prepared using the VITEK^®^ MS MOULD KIT, following the manufacturer’s protocol. Approximately 1 cm^2^ of mycelium was scraped from the agar surface with a moistened cotton bud and transferred into a 1.5 mL microtube containing 900 µL of 70% ethanol. After vortexing for 1 min and centrifugation at 16,000 g for 2 minutes, the supernatant was discarded. The pellet was then resuspended in 40 µL of 70% formic acid, vortexed, followed by the addition of 40 µL of acetonitrile and a second vortexing step. A final centrifugation at 16,000 g for 2 minutes was performed, and 1 µL of the resulting supernatant was spotted onto the target plate. CHCA matrix solution (1 µL) was added to each spot and to the calibration well, and the plate was air dried before analysis.

Automatic calibration and instrument control were performed using the *Escherichia coli* ATCC 8739 test standard. Spectra were analyzed using the VITEK^®^ MS software (version 1.1.0–203571250) with the IVD Knowledge Base (KB) version 3.2 (KB3.2) database, which was used for all PRIME identifications in this study. Confidence identification results were defined as “Single Choice” (green-coded) when the spectrum similarity level was ≥ 60% with only one match in the database, and as “Low Discrimination” (yellow-coded) when the similarity level was < 60% and/or when multiple (≤ 4) matches were retrieved. Non-identification (red-coded) was assigned when no match was found or when the system matched more than four species in the database.

**Fig. 1:**
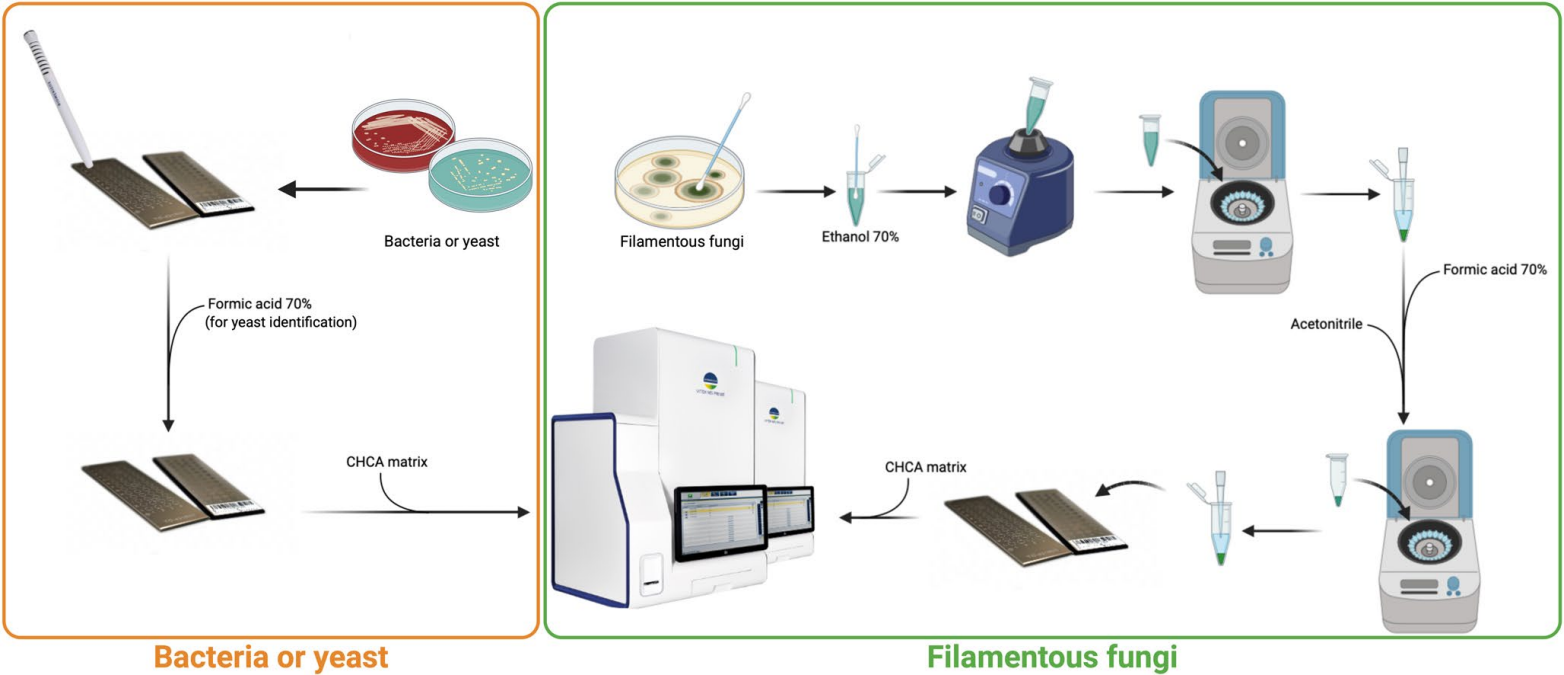
Sample preparation workflows for MALDI-TOF MS identification using the PRIME system. Bacteria and yeasts were directly spotted onto the target plate, with 70% formic acid added for yeasts, followed by CHCA matrix. Filamentous fungi underwent ethanol inactivation, extraction with formic acid and acetonitrile, and spotting on the target plate with CHCA matrix

Figure 1 illustrates the sample preparation workflow used for the identification of bacteria, yeasts, and filamentous fungi with the PRIME system.

## Biotyper system-based analysis

For bacterial isolates, direct smear preparation on the MALDI target plate was performed according to a previously published protocol [3], which reflects our routine laboratory practice but may not fully reproduce the most recent manufacturer’s instructions. For yeast isolates, a rapid protein extraction protocol was applied as described by Patel [13]: a small amount of colony material was suspended in 45 µL of distilled water and 5 µL of 70% formic acid. After mixing, 1 µL of the suspension was spotted onto the MALDI target plate and air-dried. Once dry, 1 µL of CHCA matrix solution was added to each spot.

Protein spectra were acquired using the MALDI Biotyper MBT Smart system (Bruker Daltonics) in positive ion mode with a laser frequency of 200 Hz, with each isolate analyzed in duplicate in automated mode. Each spectrum was generated from 240 laser shots (in 40 shot increments). Calibration was performed using the Bruker Bacterial Test Standard (BTS).

Spectra were analyzed using the MALDI Biotyper software (version 4.1.14) with the MBT database version 11.0.0. For the main comparative analyses, bacterial and yeast identifications were obtained using the IVD version of the MBT database. In addition, yeast isolates were also analyzed using the corresponding RUO database version to explore the impact of extended spectral coverage; these RUO-based results were considered in a secondary, descriptive analysis. Identification results were classified as high-confidence (green-coded) or low-confidence (yellow-coded) based on a log(score) value of ≥ 2.0 or between 1.7 and 1.99, respectively. Non-identification (red-coded) was assigned for log(score) values < 1.7. Given the use of literature-based protocols and different database versions (IVD and RUO), Biotyper data were used to provide a descriptive contextual comparison with PRIME rather than a formal head-to-head performance evaluation.

## PCR/sequencing-based analysis

All isolates were re-identified by targeted gene sequencing, which served as the reference method. DNA was extracted with the DNeasy Plant Mini Kit (Qiagen, Hilden, Germany); amplicons were purified with the MinElute PCR Purification Kit (Qiagen) and sequenced on a SeqStudio Genetic Analyzer (Thermo Fisher Scientific, Waltham, MA, USA).

PCR target selection, primer sets, and thermal cycling conditions followed CLSI guideline MM18 [4]. For bacteria, the 16 S rRNA gene served as the primary target, with additional loci *tuf* for Gram-positive cocci; *rpoB* or *cpn60* for Gram-negative rods; *sodA*/*gdh* for streptococci applied as needed to resolve closely related taxa.

For yeasts and molds, the internal transcribed spacer (ITS) region of the nuclear rDNA (amplified with ITS1/ITS4 primers) served as the primary target, in accordance with CLSI MM18. For yeasts, the D1/D2 domain of the large subunit (LSU) rDNA was sequenced as a reflex target when ITS was inconclusive or discordant. For filamentous fungi, sequencing of the β-tubulin (*TUB2*) or translation elongation factor 1-α (*TEF1*) gene was performed selectively to increase taxonomic resolution [1, 8].

Chromatograms were reviewed with Chromas (v2.6.6), and sequences were queried by NCBI BLAST. In accordance with CLSI MM18, identifications were accepted at ≥ 99% sequence identity for yeasts and ≥ 98% for bacteria and molds.

## Analysis of identification results

Identification results obtained with the PRIME and Biotyper systems were categorized based on the confidence levels and classification criteria described in the respective sections above. For both systems, results were stratified as correct at the species level when the identified species matched the reference identification, correct at the genus level when only the genus matched, or as misidentifications when neither genus nor species matched. Results were considered “non-identification” when the system failed to return a reliable identification.

Agreement with the reference identification was calculated and expressed as the percentage of correct identification, misidentification, or non-identification, together with 95% confidence intervals, which were calculated using the Wilson method. For bacteria and yeasts i.e., organism groups tested with both systems PRIME and Biotyper were compared using McNemar’s exact test, based on paired identification outcomes. A two-sided p value < 0.05 was considered statistically significant. Statistical analyses were performed using R and the epiR package (v2.0.61; R Foundation for Statistical Computing, Vienna, Austria).

### Workflow and turnaround time analysis

To compare workflow efficiency between the two MALDI-TOF MS systems, we prospectively evaluated turnaround time (TAT) by separately measuring hands-on sample preparation time and automated instrument time. Hands-on time was defined as the interval from retrieving the culture plates under the biological safety cabinet to completion of spotting on the MALDI target plate. Automated time included target plate handling inside the instrument, spectra acquisition, and data processing by the software. A stopwatch was used to record each component, and the total TAT was calculated as the sum of hands-on and automated times, ending when the final identification result was available. Separate measurements were obtained for bacterial/yeast samples and for filamentous fungi.

## Results

### Bacterial species identification

After excluding two off-panel species (*Pseudomonas koreensis* and *Streptococcus nidrosiense*) based on DNA sequencing results, a total of 317 bacterial isolates were analyzed using both the PRIME and Biotyper systems. Among these, 143 (45.1%) were Gram-positive and 174 (54.9%) were Gram-negative isolates. The PRIME system correctly identified 311/317 isolates (98.1%; 95% CI, 95.9–99.1), misidentified 5/317 (1.6%; 95% CI, 0.7–3.6), and failed to identify 1/317 (0.3%; 95% CI, 0.1–1.8). Misidentifications included *Clostridium innocuum* (identified as *Enterococcus hirae*), *Enterobacter kobei* (as *Enterobacter cancerogenus*), *Enterobacter roggenkampii* (as *Enterobacter asburiae*), *Staphylococcus aureus* (as *Staphylococcus epidermidis*), and *Veillonella parvula* (as *Streptococcus salivarius*). The single non-identification case involved *Salmonella enterica*.

The Biotyper system correctly identified 308/317 isolates (97.2%; 95% CI, 94.7–98.5), with 3/317 misidentifications (0.9%; 95% CI, 0.3–2.7) *Enterobacter kobei* misidentified as *Enterobacter bugandensis/roggenkampii*, and two isolates of *Enterobacter roggenkampii* misidentified as *Enterobacter kobei*. Non-identification occurred in 6/317 isolates (1.9%; 95% CI, 0.9–4.1), including *Bacillus altitudinis/pumilus*, *Bacillus cereus*, *Corynebacterium striatum*, *Enterococcus faecium*, *Klebsiella pneumoniae*, and *Salmonella enterica*.

For bacteria, McNemar’s exact test was applied to paired identification outcomes for isolates tested with both systems and did not show a statistically significant difference in correct identification rates between PRIME and Biotyper (*p* > 0.05), in line with the broadly similar bacterial performance observed descriptively.

Results are summarized in Table 1 and detailed in Table S1.

**Table 1:** Identification results by the VITEK MS PRIME (“PRIME”) and Bruker MALDI biotyper (“Biotyper”) systems for clinical bacterial and yeast isolates

	PRIME system			Biotyper system		
	No. with result/No. tested	Rate (%)	95% CI (%)	No. with result/No. tested	Rate (%)	95% CI (%)
Bacterial isolates						
Identification	311/317	98.1	95.9–99.1	308/317	97.2	94.7–98.5
Misidentification	5/317	1.6	0.7–3.6	3/317	0.9	0.3–2.7
Non-identification	1/317	0.3	0.1–1.8	6/317	1.9	0.9–4.1
Yeast isolates						
Identification	185/196	94.4	90.2–96.8	120/196^a^	61.2	54.2–67.8
Misidentification	2/196	1.0	0.3–3.6	0/196	0.0	0.0–1.9
Non-identification	9/196	4.6	2.4–8.5	76/196^a^	38.8	32.2–45.8

^a^Identification and non-identification rates differed substantially from those obtained when the “research use only” (RUO) version was used instead of “in vitro diagnostic” (IVD) version with the Biotyper system, accounting for 184/196 (93.9%) and 12/196 (6.1%), respectively. The rate of misidentification remained unchanged (0/196, 0%)

### Yeast species identification

After excluding four off-panel species (*Candida ethenolitica*, *Candida mesorugosa*, *Pichia fermentans*, and *Pichia myanmarensis*) based on DNA sequencing results, a total of 196 yeast isolates were analyzed using both the PRIME and Biotyper systems. The yeast panel included common *Candida* species (such as *C. albicans*, *C. parapsilosis*, *C. tropicalis*, and *C. glabrata*), as well as less frequent yeasts including *Cryptococcus* and *Saprochaete* species. The PRIME system correctly identified 185/196 isolates (94.4%; 95% CI, 90.2–96.8), misidentified 2/196 (1.0%; 95% CI, 0.3–3.6), and failed to identify 9/196 (4.6%; 95% CI, 2.4–8.5). Misidentifications involved *Candida dubliniensis* (identified as *Candida albicans*) and *Candida parapsilosis* (as *Candida orthopsilosis*). Non-identifications included *Candida fabianii* (2 isolates), *Candida lusitaniae* (1 isolate), *Candida nivariensis* (1 isolate), *Candida tropicalis* (2 isolates), and *Cryptococcus neoformans* (3 isolates).

For the main comparative analyses, Biotyper results obtained with the IVD database version were considered. Using the IVD database, the Biotyper system correctly identified 120/196 isolates (61.2%; 95% CI, 54.2–67.8), with no misidentifications (0/196; 0.0%; 95% CI, 0.0–1.9) and 76/196 non-identifications (38.8%; 95% CI, 32.2–45.8).

When the RUO database was used, the Biotyper system yielded 184/196 correct identifications (93.9%; 95% CI, 89.6–96.5) and 12/196 non-identifications (6.1%; 95% CI, 3.5–10.4). These non-identifications involved *Candida incospicua* (1 isolate), *Candida orthopsilosis* (1 isolate), *Candida parapsilosis* (1 isolate), *Candida utilis* (2 isolates), *Cryptococcus neoformans* (1 isolate), *Cryptococcus uniguttulatus* (1 isolate), *Kodamaea ohmeri* (1 isolate), *Saprochaete capitata* (1 isolate), and *Saprochaete clavata* (3 isolates). No misidentifications were observed with the Biotyper under this database version.

For yeasts, McNemar’s exact test applied to paired outcomes in the IVD setting showed a statistically significant difference in correct identification rates between PRIME and the Biotyper IVD version (*p* < 0.05), consistent with the higher accuracy observed for PRIME. Results are summarized in Table 1 and detailed in Table S2.

### Filamentous fungal species identification

After excluding three off-panel species (*Aspergillus parasiticus* and two *Trichophyton indotineae*) based on DNA sequencing results, a total of 130 mold isolates were analyzed using the PRIME system. Of these, 112/130 isolates (86.2%; 95% CI, 79.2–91.1) were correctly identified, 2/130 (1.5%; 95% CI, 0.4–5.4) were misidentified *Fusarium oxysporum* and *Fusarium solani*, both identified as *Rhizopus arrhizus* and 16/130 (12.3%; 95% CI, 7.7–19.1) were not identified. The non-identification cases included *Beauveria bassiana* (1 isolate), *Chaetomium globosum* (1 isolate), *Fusarium solani* (7 isolates), *Lichtheimia corymbifera* (1 isolate), *Rhizopus arrhizus* (4 isolates), *Scedosporium boydii* (1 isolate), and *Trichophyton mentagrophytes* (1 isolate). Biotyper results for molds are not reported, as the corresponding database was not available. Results are summarized in Table 2 and detailed in Table S3. A genus-level breakdown of the mold panel (e.g. *Aspergillus*, *Fusarium*, *Rhizopus*) and the corresponding identification performance is provided in Table S4.

**Table 2:** Identification results by the VITEK MS PRIME (“PRIME”) system for clinical mold isolates

	PRIME system		
	No. with result/No. tested	Rate (%)	95% CI (%)
Mold isolates^a^			
Identification	112/130	86.2	79.2–91.1
Misidentification	2/130	1.5	0.4–5.4
Non-identification	16/130	12.3	7.7–19.1

^a^Unlike bacterial and yeast isolates (see Table 1), mold isolates were analyzed exclusively using the PRIME system, as the mold database was not available in the Bruker MALDI Biotyper system employed in this study

All isolates that were correctly identified at the species level in Table S3 were also correctly assigned at the genus level in the complementary genus-level analysis (Table S4), whereas none of the isolates that were misidentified or not identified at the species level were correctly identified at the genus level.

### Workflow and turnaround time

For bacterial and yeast isolates, the average hands-on time for sample preparation was approximately 8 min with PRIME and 4 min with Biotyper. For filamentous fungi, hands-on time was longer for both systems because of the additional extraction steps, averaging about 15 min with PRIME and 15–20 min with Biotyper, depending on sample complexity.

With PRIME, automated spectra acquisition and analysis required approximately 20–30 s per sample, corresponding to a total acquisition time of about 5 min per run, partly reflecting the calibration procedure that reads the ATCC standard strain at both the beginning and the end of the acquisition. In contrast, Biotyper completed spectra acquisition in roughly 15 s per sample, with a total automated time of around 3 min per run.

Overall, the total TAT (hands-on plus automated time) for bacterial and yeast samples was approximately 13 min with PRIME and 9 min with Biotyper. For filamentous fungi, total TAT was about 20 min with PRIME and 19–24 min with Biotyper.

## Discussion

### Overall identification performance of PRIME

In this study, we evaluated PRIME using the IVD KB3.2 database (the same database available for the previous-generation VITEK MS). On a pre-identified panel of clinical isolates (characterized by targeted DNA sequencing), PRIME achieved 98.1% correct identifications for bacteria, 94.4% for yeasts, and 86.2% for molds, with a non-identification rate of 12.3% for molds. Across organism groups, non-identification was rare for bacteria and yeasts but more frequent for molds, in line with the known challenges of MALDI-TOF MS–based fungal identification [13, 18]. These aspects are further detailed in the section on fungal performance below.

For contextual comparison, bacterial and yeast isolates were also analyzed with Biotyper: using the IVD database, Biotyper achieved 97.2% correct identifications for bacteria and 61.2% for yeasts, with yeast accuracy increasing to 93.9% when the RUO database was used. Paired analyses based on McNemar’s exact test showed no significant difference between PRIME and Biotyper for bacteria, whereas PRIME outperformed Biotyper in IVD version for yeasts. When interpreting these inter-system differences, it is important to consider that PRIME was evaluated exclusively with the IVD KB3.2 database, whereas Biotyper was used with the IVD database for bacteria and with both IVD and RUO databases for yeasts. The higher yeast accuracy observed with the Biotyper RUO version mainly reflects extended library coverage rather than hardware differences, and Biotyper results in our study are therefore best regarded as a descriptive contextual comparator rather than as a formal head-to-head benchmark.

### PRIME performance for fungal isolates

Historical comparisons must account for database evolution. Using older VITEK MS IVD releases (e.g., version 2.0), Lévesque et al. [10] reported much lower mold identification, largely due to limited on-panel representation. By contrast, recent PRIME evaluations with IVD KB3.2 show clear improvement. In particular, Dutkiewicz et al. [6] and Klugherz et al. [7] both using PRIME with IVD KB3.2 reported mold identification rates of 77% and 81%, respectively: the former across 169 isolates (61 *Aspergillus*, 72 rare molds, 36 uncommon yeasts), the latter across 77 mold isolates with a more evenly distributed species set typical of clinical mycology. Our 86.2% mold identification therefore sits at the upper end of contemporary reports, likely reflecting species distribution, extraction efficiency, and expanded reference spectra. At the genus level, our complementary analysis confirmed that species-level correct identifications translated into correct genus assignment, whereas isolates that were misidentified or not identified at the species level were consistently problematic also at the genus level, underscoring the need for further database refinement for selected fungal taxa.

For yeasts, the near-equivalence between PRIME and Biotyper when the RUO database is used (94.4% vs. 93.9%) underscores that library breadth more than instrumentation governs performance for difficult or cryptic taxa. In this context, optimized extraction protocols can further assist the identification of encapsulated or thick-walled yeasts. Consistently, Dutkiewicz et al. [6], analyzing uncommon yeasts with different fungal libraries, reported that the highest rates of correctly identified isolates at both genus and species level (92%) were obtained with VITEK MS PRIME used with KB3.2 and KB3.3. Taken together, these data and our findings support the view that the main driver of fungal performance is the breadth and curation of the spectral libraries, with PRIME/KB3.2 performing at least comparably to other contemporary MALDI-TOF MS configurations.

### Misidentifications and practical implications

Misidentifications were rare for both systems and clustered in a limited number of taxa. For bacteria, PRIME misidentified 5/317 isolates and Biotyper 3/317; for yeasts, only 2/196 PRIME identifications were incorrect, and no misidentifications occurred with Biotyper; for molds, 2/130 PRIME identifications were misassigned. PRIME errors involved, among others, *Clostridium innocuum* reported as *Enterococcus hirae* and *Veillonella parvula* as *Streptococcus salivarius*, while Biotyper misclassifications were confined to species within the *Enterobacter* genus. These patterns highlight known challenging groups, including anaerobes and closely related *Enterobacter* species, where spectral similarity and uneven library coverage can affect classification.

For yeasts, both PRIME misidentifications involved well-recognized complexes *Candida parapsilosis* vs. *Candida orthopsilosis* and *Candida dubliniensis* vs. *Candida albicans* with negligible clinical impact in our setting (no change in antifungal therapy would have ensued). For molds, PRIME misclassified *Fusarium oxysporum* and *Fusarium solani* as *Rhizopus arrhizus*, and non-identification events concentrated in *F. solani*, underscoring the need for broader intraspecies representation and, in some cases, more robust extraction. In routine practice, such residual risk is best mitigated by plausibility checks integrating microscopy or Gram stain, colony morphology and growth characteristics, the clinical source, repeat extraction when results are unexpected, and recourse to sequencing for clinically critical or discordant identifications.

### Strengths and limitations

Among the strengths of this evaluation are the use of a broad and taxonomically diverse panel of clinical isolates, the inclusion of challenging organism groups such as uncommon yeasts and filamentous fungi, and the standardized reference identification by PCR and sequencing. The study also provides contextual comparison with Biotyper for bacteria and yeasts, although the focus remains on assessing PRIME performance in a routine tertiary-care laboratory.

Several limitations should be acknowledged. First, the number of isolates for some rarely encountered species was small; notably, our only two *Veillonella parvula* and *Clostridium innocuum* isolates were misidentified by PRIME, and half of the *Enterobacter kobei* (2 isolates) and *Enterobacter roggenkampii* (3 isolates) isolates were misidentified across systems. Second, database asymmetry limits direct inter-system comparisons: PRIME was evaluated exclusively with the IVD KB3.2 database, whereas Biotyper performance differed substantially between IVD and RUO versions for yeasts. As the RUO library provides broader coverage, differences between PRIME and Biotyper should therefore be interpreted descriptively rather than as formal head-to-head benchmarking.

Third, the Biotyper workflow used in this study was based on published protocols rather than manufacturer-optimized procedures, which may affect comparability [14]. Fourth, certain clinically important organism groups such as mycobacteria and actinomycetes were not included, consistent with other recent evaluations (e.g., [5]). Finally, we assessed first-pass performance only and did not implement repeat or alternative extraction methods after an initial failure, reflecting the pragmatic laboratory aim to evaluate routine throughput rather than maximal achievable yield.

## Conclusions

PRIME provided high identification accuracy for bacteria, yeasts, and molds in our routine European tertiary-care setting, with mold performance at the upper end of contemporary reports using the IVD KB3.2 database. Maximizing the diagnostic value of PRIME will require ongoing updates and curation of fungal and bacterial libraries particularly for rare or cryptic taxa together with optimized extraction protocols for difficult organisms and, when needed, integration with molecular methods to resolve ambiguous or clinically significant identifications.

## Supplementary Information

Below is the link to the electronic supplementary material.

Supplementary Material 1

Supplementary Material 2

Supplementary Material 3

Supplementary Material 4

## Data Availability

No datasets were generated or analysed during the current study.
