# Supplementary material for: Evaluation of the VITEK® MS PRIME system for routine identification of bacteria, yeasts, and molds in a tertiary care hospital laboratory
Source: Eur J Clin Microbiol Infect Dis. 2026 Mar 30;45(7):2077–84. doi: 10.1007/s10096-025-05386-0 (PMC13328275; doi:10.1007/s10096-025-05386-0)
Supplement: Supplementary file 1 — Supplementary Material 1 [file 10096_2025_5386_MOESM1_ESM.docx]

**Table S1** Identification results by the VITEK MS PRIME (“PRIME”) and Bruker MALDI Biotyper (“Biotyper”) systems for 317 clinical bacterial isolates, by species

| Species (no. of isolates tested) | PRIME results (no. isolates with result/no. isolates tested) categorized as: | | | | Biotyper results (no. isolates with result/no. isolates tested) categorized as: | | | | |
| --- | --- | --- | --- | --- | --- | --- | --- | --- | --- |
|  | Correctly identified, with level | | Misidentified | Not identified | Correctly identified, with (log)score | | Misidentified | Not identified |  |
|  | ≥60% | <60% |  |  | ≥2.0 | ≥1.7 |  |  |  |
| *Achromobacter insolitus* (1) | 1/1 |  |  |  | 1/1 |  |  |  |  |
| *Achromobacter xylosoxidans* (2) | 2/2 |  |  |  | 2/2 |  |  |  |  |
| *Acinetobacter baumannii* (22) | 22/22 |  |  |  | 20/22 | 2/22 |  |  |  |
| *Acinetobacter pittii* (1) | 1/1 |  |  |  | 1/1 |  |  |  |  |
| *Aeromonas caviae* (1) | 1/1 |  |  |  | 1/1 |  |  |  |  |
| *Bacillus altitudinis/pumilus* (1) | 1/1 |  |  |  |  |  |  | 1/1 |  |
| *Bacillus cereus* (1) | 1/1 |  |  |  |  |  |  | 1/1 |  |
| *Bacteroides fragilis* (1) | 1/1 |  |  |  | 1/1 |  |  |  |  |
| *Bacteroides ovatus* (2) | 2/2 |  |  |  | 2/2 |  |  |  |  |
| *Campylobacter jejuni* (1) | 1/1 |  |  |  | 1/1 |  |  |  |  |
| *Cardiobacterium hominis* (2) | 2/2 |  |  |  | 1/2 | 1/2 |  |  |  |
| *Citrobacter braakii* (4) | 4/4 |  |  |  | 4/4 |  |  |  |  |
| *Citrobacter freundii* (3) | 2/3 | 1/3 |  |  | 3/3 |  |  |  |  |
| *Citrobacter koseri* (4) | 4/4 |  |  |  | 4/4 |  |  |  |  |
| *Citrobacter youngae* (1) | 1/1 |  |  |  | 1/1 |  |  |  |  |
| *Clostridium innocuum* (2) | 1/2 |  | 1/2 |  | 1/2 | 1/2 |  |  |  |
| *Clostridium perfringens* (1) | 1/1 |  |  |  | 1/1 |  |  |  |  |
| *Corynebacterium amycolatum* (2) | 2/2 |  |  |  | 2/2 |  |  |  |  |
| *Corynebacterium striatum* (3) | 3/3 |  |  |  | 2/3 |  |  | 1/3 |  |
| *Corynebacterium tuberculostearicum* (1) | 1/1 |  |  |  |  | 1/1 |  |  |  |
| *Cutibacterium avidum* (1) | 1/1 |  |  |  |  | 1/1 |  |  |  |
| *Delftia acidovorans* (1) | 1/1 |  |  |  | 1/1 |  |  |  |  |
| *Enterobacter cancerogenus* (1) | 1/1 |  |  |  | 1/1 |  |  |  |  |
| *Enterobacter cloacae* (3) | 3/3 |  |  |  | 3/3 |  |  |  |  |
| *Enterobacter hormaechei* (4) | 3/4 | 1/4 |  |  | 4/4 |  |  |  |  |
| *Enterobacter kobei* (2) | 1/2 |  | 1/2 |  | 1/2 |  | 1/2 |  |  |
| *Enterobacter roggenkampii* (3) | 2/3 |  | 1/3 |  | 1/3 |  | 2/3 |  |  |
| *Enterococcus casseliflavus* (2) | 2/2 |  |  |  | 2/2 |  |  |  |  |
| *Enterococcus faecalis* (8) | 8/8 |  |  |  | 8/8 |  |  |  |  |
| *Enterococcus faecium* (13) | 13/13 |  |  |  | 11/13 | 1/13 |  | 1/13 |  |
| *Escherichia coli* (29) | 29/29 |  |  |  | 27/29 | 2/29 |  |  |  |
| *Haemophilus influenzae* (3) | 2/2 |  |  |  | 2/2 |  |  |  |  |
| *Haemophilus parainfluenzae* (1) | 1/1 |  |  |  | 1/1 |  |  |  |  |
| *Hafnia alvei* (1) |  | 1/1 |  |  | 1/1 |  |  |  |  |
| *Klebsiella aerogenes* (4) | 4/4 |  |  |  | 4/4 |  |  |  |  |
| *Klebsiella oxytoca* (3) | 3/3 |  |  |  | 3/3 |  |  |  |  |
| *Klebsiella pneumoniae* (23) | 22/23 | 1/23 |  |  | 17/23 | 5/23 |  | 1/23 |  |
| *Lactobacillus acidophilus*/*gasseri* (4) | 4/4 |  |  |  | 4/4 |  |  |  |  |
| *Lactobacillus crispatus* (2) | 2/2 |  |  |  | 2/2 |  |  |  |  |
| *Lactobacillus iners* (3) | 2/2 |  |  |  | 2/2 |  |  |  |  |
| *Lactobacillus jensenii* (1) | 1/1 |  |  |  | 1/1 |  |  |  |  |
| *Limosilactobacillus reuteri* (1) | 1/1 |  |  |  | 1/1 |  |  |  |  |
| *Listeria monocytogenes* (11) | 11/11 |  |  |  | 11/11 |  |  |  |  |
| *Micrococcus luteus* (2) | 2/2 |  |  |  | 1/2 | 1/2 |  |  |  |
| *Morganella morganii* (6) | 6/6 |  |  |  | 6/6 |  |  |  |  |
| *Neisseria flavescens* (2) | 2/2 |  |  |  | 2/2 |  |  |  |  |
| *Neisseria meningitidis* (1) | 1/1 |  |  |  | 1/1 |  |  |  |  |
| *Neisseria sicca* (1) | 1/1 |  |  |  | 1/1 |  |  |  |  |
| *Peptostreptococcus anaerobius* (1) | 1/1 |  |  |  | 1/1 |  |  |  |  |
| *Proteus mirabilis* (11) | 11/11 |  |  |  | 11/11 |  |  |  |  |
| *Proteus vulgaris* (1) | 1/1 |  |  |  | 1/1 |  |  |  |  |
| *Providencia stuartii* (2) | 2/2 |  |  |  | 2/2 |  |  |  |  |
| *Pseudomonas aeruginosa* (16) | 16/16 |  |  |  | 15/16 | 1/16 |  |  |  |
| *Raoultella ornithinolytica* (1) | 1/1 |  |  |  | 1/1 |  |  |  |  |
| *Raoultella planticola* (1) | 1/1 |  |  |  | 1/1 |  |  |  |  |
| *Rothia kristinae* (1) | 1/1 |  |  |  | 1/1 |  |  |  |  |
| *Rothia mucilaginosa* (1) | 1/1 |  |  |  |  | 1/1 |  |  |  |
| *Salmonella enterica* (4) | 3/4 |  |  | 1/4 | 2/4 | 1/4 |  | 1/4 |  |
| *Schaalia turicensis* (1) | 1/1 |  |  |  | 1/1 |  |  |  |  |
| *Serratia marcescens* (2) | 2/2 |  |  |  | 2/2 |  |  |  |  |
| *Staphylococcus aureus* (7) | 6/7 |  | 1/7 |  | 6/7 | 1/7 |  |  |  |
| *Staphylococcus capitis* (2) | 2/2 |  |  |  | 2/2 |  |  |  |  |
| *Staphylococcus epidermidis* (18) | 18/18 |  |  |  | 16/18 | 2/18 |  |  |  |
| *Staphylococcus haemolyticus* (7) | 7/7 |  |  |  | 3/7 | 4/7 |  |  |  |
| *Staphylococcus hominis* (8) | 8/8 |  |  |  | 8/8 |  |  |  |  |
| *Staphylococcus lugdunensis* (3) | 3/3 |  |  |  | 2/3 | 1/3 |  |  |  |
| *Staphylococcus pettenkoferi* (1) | 1/1 |  |  |  | 1/1 |  |  |  |  |
| *Staphylococcus warneri* (1) | 1/1 |  |  |  | 1/1 |  |  |  |  |
| *Stenotrophomonas maltophilia* (2) | 2/2 |  |  |  | 2/2 |  |  |  |  |
| *Streptococcus agalactiae* (10) | 10/10 |  |  |  | 10/10 |  |  |  |  |
| *Streptococcus anginosus* (1) | 1/1 |  |  |  | 1/1 |  |  |  |  |
| *Streptococcus constellatus* (1) | 1/1 |  |  |  | 1/1 |  |  |  |  |
| *Streptococcus gallolyticus* (2) | 2/2 |  |  |  | 2/2 |  |  |  |  |
| *Streptococcus mitis*/*oralis* (2) | 2/2 |  |  |  | 1/2 | 1/2 |  |  |  |
| *Streptococcus parasanguinis* (2) | 2/2 |  |  |  | 2/2 |  |  |  |  |
| *Streptococcus pneumoniae* (3) | 3/3 |  |  |  | 3/3 |  |  |  |  |
| *Streptococcus pyogenes* (12) | 12/12 |  |  |  | 12/12 |  |  |  |  |
| *Veillonella parvula* (1) |  |  | 1/1 |  | 1/1 |  |  |  |  |
| Total species (317)^a^ | 307/317 | 4/317 | 5/317 | 1/317 | 280/317 | 28/317 | 3/317 | 6/317 |  |

^a^Two additional isolates—*Pseudomonas koreensis* and *Streptococcus nidrosiense*—were included in the initial study set but excluded from the analysis, as they represented off-panel species for the PRIME database (in the case of *P. koreensis*) or for both the PRIME and Biotyper databases (in the case of *S. nidrosiense*).
