# Supplementary material for: Evaluation of the VITEK® MS PRIME system for routine identification of bacteria, yeasts, and molds in a tertiary care hospital laboratory
Source: Eur J Clin Microbiol Infect Dis. 2026 Mar 30;45(7):2077–84. doi: 10.1007/s10096-025-05386-0 (PMC13328275; doi:10.1007/s10096-025-05386-0)
Supplement: Supplementary file 2 — Supplementary Material 2 [file 10096_2025_5386_MOESM2_ESM.docx]

**Table S2** Identification results by the VITEK MS PRIME (“PRIME”) and Bruker MALDI Biotyper (“Biotyper”) systems for 196 clinical yeast isolates, by species

| Species (no. of isolates tested) | PRIME results (no. isolates with result/no. isolates tested) categorized as: | | | | Biotyper results (no. isolates with result/no. isolates tested) categorized, as described below, using the | | | | | | | |
| --- | --- | --- | --- | --- | --- | --- | --- | --- | --- | --- | --- | --- |
|  |  |  |  |  | IVD database | | | | RUO database | | | |
|  | Identified, with level | | Misidentified | Not identified | Identified, with (log)score | | Misidentified | Not identified | Identified, with (log)score | | Misidentified | Not identified |
|  | ≥60% | <60% |  |  | ≥2.0 | ≥1.7 |  |  | ≥2.0 | ≥1.7 |  |  |
| *Candida albicans* (23) | 23/23 |  |  |  | 7/23 |  |  | 16/23 | 23/23 |  |  |  |
| *Candida auris* (16) | 16/16 |  |  |  | 14/16 | 2/16 |  |  | 16/16 |  |  |  |
| *Candida dubliniensis* (5) | 4/5 |  | 1/5 |  | 2/5 |  |  | 3/5 | 5/5 |  |  |  |
| *Candida fabianii* (3) | 1/3 |  |  | 2/3 |  | 2/3 |  | 1/3 | 3/3 |  |  |  |
| *Candida glabrata* (15) | 15/15 |  |  |  | 11/15 |  |  | 4/15 | 15/15 |  |  |  |
| *Candida guilliermondii* (6) | 6/6 |  |  |  | 6/6 |  |  |  | 5/6 | 1/6 |  |  |
| *Candida incospicua* (1) | 1/1 |  |  |  |  |  |  | 1/1 |  |  |  | 1/1 |
| *Candida kefyr* (5) | 5/5 |  |  |  | 2/5 | 3/5 |  |  | 5/5 |  |  |  |
| *Candida krusei* (10) | 10/10 |  |  |  | 2/10 | 1/10 |  | 7/10 | 10/10 |  |  |  |
| *Candida lusitaniae* (7) | 6/7 |  |  | 1/7 | 4/7 | 3/7 |  |  | 5/7 | 2/7 |  |  |
| *Candida metapsilosis* (6) | 5/6 | 1/6 |  |  | 1/6 | 1/6 |  | 4/6 | 6/6 |  |  |  |
| *Candida nivariensis* (1) |  |  |  | 1/1 |  |  |  | 1/1 | 1/1 |  |  |  |
| *Candida norvegensis* (2) | 2/2 |  |  |  | 2/2 |  |  |  | 1/2 | 1/2 |  |  |
| *Candida orthopsilosis* (10) | 10/10 |  |  |  |  | 4/10 |  | 6/10 | 9/10 |  |  | 1/10 |
| *Candida parapsilosis* (24) | 22/24 | 1/24 | 1/24 |  | 9/24 | 5/24 |  | 10/24 | 22/24 | 1/24 |  | 1/24 |
| *Candida pararugosa* (1) | 1/1 |  |  |  |  | 1/1 |  |  | 1/1 |  |  |  |
| *Candida tropicalis* (15) | 12/15 | 1/15 |  | 2/5 | 8/15 | 1/15 |  | 6/15 | 14/15 | 1/15 |  |  |
| *Candida utilis* (3) | 3/3 |  |  |  | 2/3 | 1/3 |  |  | 1/3 |  |  | 2/3 |
| *Cryptococcus neoformans* (8) | 5/8 |  |  | 3/8 | 1/8 | 4/8 |  | 3/8 | 7/8 |  |  | 1/8 |
| *Cryptococcus uniguttulatus* (1) | 1/1 |  |  |  |  |  |  | 1/1 |  |  |  | 1/1 |
| *Kodamaea ohmeri* (1) | 1/1 |  |  |  |  |  |  | 1/1 |  |  |  | 1/1 |
| *Rhodotorula mucilaginosa* (2) | 2/2 |  |  |  | 1/2 | 1/2 |  |  | 2/2 |  |  |  |
| *Saccharomyces cerevisiae* (6) | 6/6 |  |  |  |  |  |  | 6/6 | 6/6 |  |  |  |
| *Saprochaete capitata* (8) | 8/8 |  |  |  | 7/8 | 1/8 |  |  | 7/8 |  |  | 1/8 |
| *Saprochaete clavata* (3) | 3/3 |  |  |  |  |  |  | 3/3 |  |  |  | 3/3 |
| *Trichosporon asahii* (14) | 14/14 |  |  |  | 6/14 | 5/14 |  | 3/14 | 11/14 | 3/14 |  |  |
| Total species (196)^a^ | 180/196 | 3/196 | 2/196 | 9/196 | 85/196 | 35/196 |  | 76/196 | 175/196 | 9/196 |  | 12/196 |

^a^Four additional isolates—*Candida ethenolitica*, *Candida mesorugosa*, *Pichia fermentans*, and *Pichia myanmarensis* —were included in the initial study set but excluded from the analysis, as they represented off-panel species for the PRIME database (in the case of *C. mesorugosa* and *P. fermentans*) or for both the PRIME and Biotyper databases (in the case of *C. ethenolitica* and *P. myanmarensis*).
