# Supplementary material for: Evaluation of the VITEK® MS PRIME system for routine identification of bacteria, yeasts, and molds in a tertiary care hospital laboratory
Source: Eur J Clin Microbiol Infect Dis. 2026 Mar 30;45(7):2077–84. doi: 10.1007/s10096-025-05386-0 (PMC13328275; doi:10.1007/s10096-025-05386-0)
Supplement: Supplementary file 3 — Supplementary Material 3 [file 10096_2025_5386_MOESM3_ESM.docx]

**Table S3** Identification results by the VITEK MS PRIME (“PRIME”) system for 130 clinical mold isolates, by species

| Species (no. of isolates tested) | PRIME results (no. isolates with result/no. isolates tested) categorized as: | | | |
| --- | --- | --- | --- | --- |
|  | Identified, with level | | Misidentified | Not identified |
|  | ≥60% | <60% |  |  |
| *Aspergillus flavus* (5) | 5/5 |  |  |  |
| *Aspergillus fumigatus* (13) | 13/13 |  |  |  |
| *Aspergillus intermedius* (1) | 1/1 |  |  |  |
| *Aspergillus lentulus* (1) | 1/1 |  |  |  |
| *Aspergillus nidulans* (1) | 1/1 |  |  |  |
| *Aspergillus niger* (4) | 4/4 |  |  |  |
| *Aspergillus oryzae* (3) | 3/3 |  |  |  |
| *Aspergillus sydowii* (2) | 2/2 |  |  |  |
| *Aspergillus tubingensis* (1) | 1/1 |  |  |  |
| *Aspergillus terreus* (7) | 7/7 |  |  |  |
| *Beauveria bassiana* (1) |  |  |  | 1/1 |
| *Chaetomium globosum* (1) |  |  |  | 1/1 |
| *Epicoccum nigrum* (1) | 1/1 |  |  |  |
| *Fusarium equiseti* (2) | 2/2 |  |  |  |
| *Fusarium oxysporum* (15) | 14/15 |  | 1/15 |  |
| *Fusarium proliferatum* (3) | 3/3 |  |  |  |
| *Fusarium solani* (21) | 13/21 |  | 1/21 | 7/21 |
| *Fusarium sporotrichioides* (1) | 1/1 |  |  |  |
| *Fusarium verticillioides* (3) | 3/3 |  |  |  |
| *Gibberella intermedia* (1) | 1/1 |  |  |  |
| *Lichtheimia corymbifera* (4) | 3/4 |  |  | 1/4 |
| *Mucor circinelloides* (4) | 4/4 |  |  |  |
| *Mucor racemosus* (1) | 1/1 |  |  |  |
| *Purpureocillium lilacinum* (1) | 1/1 |  |  |  |
| *Rhizopus arrhizus* (12) | 6/12 | 2/12 |  | 4/12 |
| *Scedosporium apiospermum* (2) | 2/2 |  |  |  |
| *Scedosporium boydii* (1) |  |  |  | 1/1 |
| *Trichophyton erinacei* (8) | 1/1 |  |  |  |
| *Trichophyton interdigitale* (8) | 8/8 |  |  |  |
| *Trichophyton mentagrophytes* (7) | 7/7 |  |  |  |
| *Trichophyton tonsurans* (1) | 1/1 |  |  | 1/8 |
| Total species (130)^a^ | 110/130 | 2/130 | 2/130 | 16/130 |

^a^Three additional mold isolates—one *Aspergillus parasiticus* and two *Trichophyton indotineae*—were included in the initial study set but excluded from the analysis, as they represented off-panel species for the PRIME database.
