# Supplementary material for: Evaluation of the VITEK® MS PRIME system for routine identification of bacteria, yeasts, and molds in a tertiary care hospital laboratory
Source: Eur J Clin Microbiol Infect Dis. 2026 Mar 30;45(7):2077–84. doi: 10.1007/s10096-025-05386-0 (PMC13328275; doi:10.1007/s10096-025-05386-0)
Supplement: Supplementary file 4 — Supplementary Material 4 [file 10096_2025_5386_MOESM4_ESM.docx]

**Table S4** Identification results by the VITEK MS PRIME (“PRIME”) system for 130 clinical mold isolates, by genus

| Group (no. of isolates tested) | PRIME results (no. isolates with result/no. isolates tested) categorized as: |  |  |
| --- | --- | --- | --- |
|  | Identified | Misidentified | Not identified |
| *Aspergillus* species (38) | 38/38 |  |  |
| *Fusarium* species (45) | 36/45 | 2/45 | 7/45 |
| Mucorales species (21)^a^ | 16/21 |  | 5/21 |
| *Scedosporium* species (3) | 2/3 |  | 1/3 |
| *Trichophyton* species (18) | 17/18 |  | 1/18 |
| Other molds (5)^b^ | 3/5 |  | 2/5 |
| Total molds (130)^c^ | 112/130 | 2/130 | 16/130 |

^a^Including *Lichtheimia* (n = 4), *Mucor* (n = 5), and *Rhizopus* (n = 12) species.

^b^Including uncommon molds; the two isolates that were not identified at the genus level belonged to *Beauveria bassiana* (n = 1) and *Chaetomium globosum* (n = 1).

^c^Three additional mold isolates—one *Aspergillus parasiticus* and two *Trichophyton indotineae*—were included in the initial study set but excluded from the analysis, as they represented off-panel species for the PRIME database.
